# PReS-FINAL-2006: Resilience and coping in adolentes with rheumatic illness

**DOI:** 10.1186/1546-0096-11-S2-P19

**Published:** 2013-12-05

**Authors:** M Alessio, F Fontana, A Officioso, S Cotini, F Fontana, C Forni, M Alessio

**Affiliations:** 1 Policlinico Federico II Napoli, Italy; 2 Policlinico Federico II, Naples, Italy

## Introduction

The adolescents with rheumatic disease are not only suffering physically, but they are also in the midst of an intense emotional experience, for that is important to help patients to bounce back from the double challenge, illness and adolescence.

## Objectives

In this study, we aim to assess resilience and to analyze the resilience styles and the coping skills of the subjects within the care pathway trying to obtain the helpful feedback.

## Methods

The reported studies involved rheumatic patients (n = 22 out of them 8 males) aged 15,68 + 1,08 years at 4 years from the onset of their illness.

The applied tools included the Coping Inventory for Stressful Situations (CISS) which measures coping with stress style understood as a trait of personality and the Italian version of the (RPQ) Resilience Process Questionnaire.

## Results

Among these adolescents with rheumatic disease, resilience was significantly related to the avoidance-oriented coping (P < 0.05). See table 1

**Table 1:** 

RPQ	Dysfunctional Reintegration Resilient Reintegration Return to baseline/Homeostasis	3,81 ± 1,56 5,0 ± 1,77 7,36 ± 1,43	N.V < 8 N.V > 8 N.V > 8
**CISS**	Task coping Emotion coping Avoidance coping	51,13 ± 8,19 46,56 ± 9,0 78,73 ± 19,5	N.V 45-55 N.V 45-55 N.V 45-55

## Conclusion

The results of this study shows that our patients have the ability to withstand stressful events, but their strategies do not lead to a positive resolution, since the resilience appears to be low and they have avoidance coping strategies.

The results of the presented study may become a stimulus to creating prevention.

## Disclosure of interest

None declared.

